# Relationships between implicit motives, self-attributed motives, and personal goal commitments

**DOI:** 10.3389/fpsyg.2013.00923

**Published:** 2013-12-09

**Authors:** Maika Rawolle, Maria Schultheiss, Oliver C. Schultheiss

**Affiliations:** ^1^Chair of Psychology, TUM School of Management, Technische Universität MünchenMunich, Germany; ^2^Chair for Experimental Psychology, Motivation, and Affective Neuroscience, Department of Psychology and Sport Sciences, Friedrich-Alexander UniversityErlangen, Germany

**Keywords:** implicit motives, self-attributed motives, personal goals, picture story exercise, culture, relationships

## Abstract

This research examined the relationships between measures of the implicit and the explicit motivational systems. We analyzed the relationships between picture-story measures of implicit motives, questionnaire measures of self-attributed motives, and ideographically assessed personal goal commitments within the domains achievement, affiliation, and power through a reanalysis of three data sets from the USA and Germany (total *N* = 309). No significant positive within-domain correlations of implicit motives with self-attributed motives or personal goal commitments were found, and self-attributed motives correlated substantially and positively with personal goals. Results did not systematically differ between data sets.

## Introduction

McClelland et al. (1989) proposed the existence of conscious (i.e., explicit) and nonconscious (i.e., implicit) motivation systems. The implicit system consists of a limited number of implicit motives, that is, associative networks in the mid-brain (McClelland et al., 1989; Weinberger and McClelland, 1990) that constitute motivational needs (i.e., the needs for power, affiliation, and achievement, abbreviated as n Power, n Affiliation, etc.) and represent capacities to experience particular types of incentives as pleasurable or aversive (Schultheiss, 2008). Implicit motives develop preverbally through affective experiences (McClelland and Pilon, 1983). The explicit system, in contrast, houses self-attributed motives, that is, language-based representations of individuals' beliefs about their motivational orientations, and their verbally represented personal goals, that is, cognitive representations of what a person currently wants to achieve (Brunstein et al., 1998). Self-attributed motives as well as personal goals develop later in life through verbally encoded experiences.

As a consequence of their differences in development, neuro-physiological representation, and cognitive elaboration, the implicit and the explicit motivational systems system are thought to be distinct (McClelland et al., 1989). This assumption has received empirical support in the sense that measures tapping the implicit system do not correlate substantially with measures tapping the explicit system (e.g., Spangler, 1992; King, 1995; Schultheiss and Brunstein, 2001; Pang and Schultheiss, 2005). Beyond these correlative studies, there is a large number of experimental studies documenting the functional independence of these systems in terms of the types of incentives they respond to and the types of behavior they predict (e.g., deCharms et al., 1955; McClelland, 1985; Koestner et al., 1991; Brunstein and Maier, 2005; see Spangler, 1992, for a meta-analysis of functional dissociation effects in the domain of n Achievement). A further characteristic of implicit motives is that they are not consciously accessible and hence need to be measured through indirect means like the Picture Story Exercise (PSE; McClelland et al., 1989). Self-attributed motives and personal goals, in contrast, are consciously accessible and can therefore be measured via self-report techniques (e.g., Personality Research Form, PRF; Jackson, 1984; or the Personal Goal Inventory, PGI, Brunstein et al., 1998). The distinctiveness of the two systems implies that individuals vary widely in the degree of between-system alignment. Recent research shows that certain personality traits, such as self-determination (Thrash and Elliot, 2002; Hofer et al., 2010a), referential competence (Schultheiss et al., 2011), private body-consciousness, preference for consistency and a low tendency for self-monitoring (Thrash et al., 2007), promote a person's implicit-explicit-alignment and that this alignment has beneficial effects on well-being (Brunstein et al., 1998; Hofer and Chasiotis, 2003; Baumann et al., 2005; Hofer et al., 2006; Schultheiss et al., 2008a; for a summary see Thrash et al., 2012).

The notion that implicit motives do not converge much with self-attributed motives and personal goals (McClelland et al., 1989), whereas the latter, as parts of the same system, are associated with each other (Weinberger and McClelland, 1990), is so deeply entrenched in the literature that, despite being the theoretical basis for many studies and also being occasionally challenged (e.g., Thrash et al., 2010), to date only two studies have tested it by simultaneously measuring all three constructs and examining their relationships. However, these studies yielded conflicting results (Emmons and McAdams, 1991; King, 1995; see below). In the present research, we simultaneously assessed all three constructs in three studies to provide further clarification of their relationships. In the following, we start with a brief overview of prior research presenting the relationships between implicit motives and self-attributed motives, between implicit motives and personal goals, as well as between self-attributed motives and personal goals, respectively.

Although some studies occasionally find significant positive correlations between measures of implicit motives and self-attributed motives (e.g., Thrash and Elliot, 2002), many others do not (e.g., King, 1995; Pang and Schultheiss, 2005). Meta-analytic findings also suggest that the variance overlap between implicit and explicit motive measures is less than 1% (Spangler, 1992). Even if self-report measures are matched to PSE measures by modeling items after the PSE content categories (Thrash et al., 2007) and presenting them in the context of the PSE pictures (Schultheiss et al., 2009), the variance overlap does not exceed 3%. These findings corroborate McClelland et al.'s (1989) claim that implicit and explicit motive measures tap into two distinct levels of human motivation.

Traditional goal theories assume that motives, whether implicit or self-attributed, guide behavior by influencing the adoption of goals (e.g., Murray, 1938; Elliot and Church, 1997; Elliot and Sheldon, 1997). In support of this prediction, Emmons and McAdams (1991) report that implicit motives and personal goals within the same motive domain are moderately correlated (*r*s ~0.40; *N* = 72). Elliot et al. (1999) as well as Thrash and Elliot (2002) report similar results for the domain of achievement (*r*s ~0.20). However, according to McClelland's model, personal goals do not reflect the person's implicit motives, because the former orient behavior toward temporally distant end states represented in language whereas the latter respond to the nonverbal incentives present in a given situation (McClelland et al., 1989; Weinberger and McClelland, 1990). And indeed, empirical studies often fail to find support for substantial positive association between the constructs. King (1995) used the same measurement approach as Emmons and McAdams (1991) in a study with a larger sample (*N* = 101) and therefore better statistical power. However, she found no significant correlations between implicit motive and explicit goal measures and thus failed to replicate the findings reported by Emmons and McAdams (1991). Correlations between implicit motive and explicit goal measures in other studies typically range from less than 0.00 to 0.20 (e.g., King, 1995; Brunstein et al., 1998; Hofer and Chasiotis, 2003; Schultheiss et al., 2008a). This suggests that the findings reported by Emmons and McAdams (1991) and by Elliot et al. (1999); Thrash and Elliot (2002) represent exceptions, not the rule. Moreover, recent studies by Hofer et al. (e.g., Hofer and Chasiotis, 2003; Hofer et al., 2006) suggest that the lacking overlap between the implicit and the explicit system is valid across cultures.

The most common view regarding the relation between personal goals and self-attributed motives is that both represent a part of the explicit system: a person's self-attributed motives guide which goals the person chooses (cf. Hofer et al., 2010a,b). Hence, despite being distinct constructs, they should be functionally related. This notion has received support in various studies, in which self-attributed motives were measured via PRF and personal goals either via a self-generated lists of goals which were content-coded for their dominant motivational theme (Emmons and McAdams, 1991; King, 1995) or a list of typical power-, achievement, and affiliation-related goals which participants judged according to how much they endorsed them (Elliot and Church, 1997; Hofer et al., 2010b). These studies yielded weak to moderate correlations between measures of self-attributed motives and personal goals.

To conclude, the relationships between measures of implicit motives on the one hand and self-attributed motives and personal goals on the other have been examined in a number of studies which paint a somewhat inconsistent picture. Moreover, only two studies have assessed all three constructs simultaneously (cf. Emmons and McAdams, 1991; King, 1995). These studies, despite using similar assessment methods, yielded inconsistent results regarding the relationship between implicit motives and personal goals. Emmons and McAdams' (1991) study yields a strong positive relationship, whereas King (1995) found no significant overlap between implicit motive and goal striving measures.

Since then, no study has attempted to assess all three constructs. Hence, existing research is based on a patch-work of rather inconsistent results. Further, none of the previous studies has examined relationships between measures cross-culturally. In this study, we aim to shed further light on the relationships between implicit motives, self-attributed motives, and personal goals as well as to test them across culturally divergent samples.

Based on McClelland et al.'s (1989) proposal of two autonomously operating motivation systems, we hypothesize that in all motive domains (1) explicit measures (i.e., self-attributed motives, personal goals) show a very low overlap with implicit measures (i.e., PSE); whereas (2) personal goals and self-attributed motives show more substantial and positive variance overlap. Further—based on the idea that the above-mentioned differences between implicit and explicit systems should be universal—we (3) expect to see these predicted correlation patterns in both Germans and US Americans. We tested these hypotheses through a reanalysis of data from three studies conducted in the United States and Germany.

## Methods

### Overview

Data were collected in two cross-sectional studies (Studies 1 and 2) conducted at the University of Michigan, Ann Arbor, USA, between 2005 and 2007, and one cross-sectional study (Study 3) conducted at Friedrich-Alexander University, Erlangen, Germany, in 2008. Findings from Study 1 on the interplay between implicit motives and personal goals on emotional well-being and depression have been reported by Schultheiss et al. (2008a; Study 2). Findings from Study 3 pertaining to associations between progesterone and indices of the coherence of cognitive functions have been reported by Schultheiss et al. (2012). Participants in the US studies received course credit; German students were paid.

### Participants

Study 1 consists of 98 participants (47 women; mean age = 21 years), Study 2 of 112 participants (59 women; mean age = 19 years) and Study 3 of 99 participants (49 women, mean age = 23 years). Thus, the combined data set consists of 309 participants.

### Procedure

In all three studies, we first assessed implicit motives, then personal goals, and finally self-attributed motives.

### Implicit motives

In Studies 1 and 3, implicit motives were assessed by having participants write an imaginative story about six pictures: ship captain, couple by river, trapeze artists, women in laboratory, boxer, and nightclub scene. In Study 2, two additional pictures were included: bicycle race and girlfriends in cafe with male approaching. Pang and Schultheiss (2005) and Schultheiss et al. (2009) provide descriptive and validation data for these 6-picture and 8-picture PSEs^1^, respectively. Pictures were presented in random order, using standard instructions and procedures described in Smith et al. (1992). For each study, stories were coded for motivational imagery by a trained scorer using the manual of Winter (1994). In Study 3, stories were coded by two independent scorers and scores were averaged across scorers for further analyses. Scorers had previously exceeded 85% interrater agreement on calibration materials prescored by an expert and were blind with regard to participants' gender and scores on the other measures. Because longer protocols were associated with more imagery for each motive, we regressed word count sum scores from each of the motive imagery sum scores within each study.

### Self-attributed motives

In Study 1, self-attributed motives in the domains of power, achievement, and affiliation were assessed with the scales social potency (e.g., “I am a natural leader; others defer to me”), achievement (e.g., “I am extremely hardworking”), and social closeness (e.g., “I am extremely affectionate. I value close personal relationships”) from the Iowa Personality Questionnaire (IPQ; Donnellan et al., 2005). Each scale consisted of three items with a biploar 5-point scale. Donnellan et al. (2005) report Cronbach's alphas between 0.57 and 0.68 for these scales.

In Studies 2 and 3, self-attributed motives were assessed with the scales dominance (for power; e.g., “The ability to be a leader is very important to me”), achievement (e.g., “I enjoy difficult work”), and affiliation (e.g., “I try to be in the company of friends as much as possible”) of the PRF [(Jackson, 1984); German version by Stumpf et al. (1985)]. Each subscale included 16 True/False (1/0) questions that describe habits consistent or inconsistent with each motivational domain. Participants were asked to decide how representative each statement was as a self-description.

### Personal goal commitments

Participants' personal goals within the domains affiliation, achievement, and power were assessed with Brunstein et al.'s Personal Goal Inventory^1^ (PGI; 1998). Participants were asked to list and describe one ideographic goal within each of three domains: (a) “striving for affiliation and friendly social contacts” (affiliation), (b) “striving for achievement and mastery experiences” (achievement) and (c) “striving for independence, social influence, and self-reliance” (power). Each area was illustrated by examples adopted from pilot work. All participants listed three goals, one goal for each area. Subsequently, they rated each goal on a 4-item scale assessing their commitment (e.g., “No matter what happens, I will not give up this goal”). Response scales ranged from 1 (*disagree strongly*) to 5 (*agree strongly*). Goal commitment items were summed within each of the three domains. In contrast to Emmons and McAdams' (1991) technique, this measure does not use the same coding system that is used for measuring implicit motives to assess explicit goal content and thus avoids method overlap.

### Data preparation

Data collection procedures and instruments differed slightly between the three studies (e.g., self-attributed motives are measured via IPQ in Study 1 vs. PRF in Studies 2 and 3; a longer PSE in Study 2 than in the other studies). We therefore converted all scores within each study to z scores before combining samples into a joint data set to bring all measures into the same meaningful metric.

## Results

Descriptive statistics and internal consistencies are presented in Table 1. As internal consistency measures are not suitable for gauging the reliability of the PSE (see Schultheiss et al., 2008b), we present the reliability between PSE-rater and expert as the index of concordance (Study 1 and 2) or the inter-rater reliability (Intraclass Correlation Coefficient, ICC; cf., Shrout and Fleiss, 1979) of two PSE-raters (Study 3). To compute the ICCs, we used a two-way random single measures model (with absolute agreement definition) where the effects due to coders, to the interaction of coders and PSE-protocols, and to random error cannot be separated. ICC values greater than 0.74 indicate excellent reliability, values from 0.60 to 0.74 are considered good [cf. Meyer et al. (2002)].

**Table 1:** **Descriptive statistics and reliabilities for each scale and sample**.

**Study**	**Variable**	**Mean**	***SD***	**Reliability**
1	IPQ-Achievement	11.85	2.17	0.72
	IPQ-Social Potency	10.32	2.41	0.69
	IPQ-Social Closeness	10.46	2.65	0.65
	PGI-Affiliation	3.60	0.87	0.80
	PGI-Achievement	4.20	0.75	0.75
	PGI-Power	3.93	0.76	0.76
	PSE-Affiliation	6.12	2.60	0.85
	PSE-Achievement	4.90	2.67	0.85
	PSE-Power	5.52	2.73	0.85
2	PRF-Achievement	10.57	3.11	0.69
	PRF-Affiliation	11.46	3.09	0.74
	PRF-Dominance	9.63	3.42	0.76
	PGI-Affiliation	3.46	0.86	0.74
	PGI-Achievement	4.14	0.72	0.75
	PGI-Power	3.49	0.88	0.73
	PSE-Affiliation	7.14	3.30	0.85
	PSE-Achievement	6.54	2.93	0.85
	PSE-Power	5.19	3.15	0.85
3	PRF-Achievement	10.86	2.97	0.69
	PRF-Affiliation	11.77	3.38	0.80
	PRF-Dominance	9.34	3.88	0.81
	PGI-Affiliation	3.88	0.78	0.76
	PGI-Achievement	4.02	0.75	0.76
	PGI-Power	3.88	0.78	0.86
	PSE-Affiliation	6.56	2.88	0.87
	PSE-Achievement	5.01	2.48	0.77
	PSE-Power	3.87	2.40	0.65

The reliability for PSE scores represents reliability between scorer and expert (index of concordance; Studies 1 and 2) or the inter-rater reliability of two PSE scorers (ICCs; Study 3).

Age and gender had no significant influence on the between-measure correlations reported below.

To test our hypotheses regarding the relationships between implicit motives, self-attributed motives and personal goal commitments within a given domain (power, achievement, or affiliation), we first computed Pearson correlations with standardized scores (see Table 2).

**Table 2:** **Correlations between personal goal commitments (PGI), self-attributed motives (PRF/IPQ), and implicit motives (PSE)**.

**Variable**	**1**	**2**	**3**	**4**	**5**	**6**	**7**	**8**	**9**
1. PGI Power	–								
2. PGI Achievement	0.26^***^	–							
3. PGI Affiliation	0.56^***^	0.26^***^	–						
4. PRF/IPQ Power	0.15^**^ (0.07)	0.15^**^	0.13^*^	–					
5. PRF/IPQ Achievement	0.14^*^	0.31^***^ (0.26^***^)	0.11	0.30^***^	–				
6. PRF/IPQ Affiliation	0.12^*^	0.03	0.26^***^ (0.24^***^)	0.27^***^	0.15^**^	–			
7. PSE Power	−0.12^*^ (−0.13^*^)	−0.08	−0.00	**−0.00 (0.02)**	0.01	−0.12^*^	–		
8. PSE Achievement	0.11	**0.04 (0.01)**	0.13^*^	−0.01	**−0.01 (−0.03)**	0.04	0.13^*^	–	
9. PSE Affiliation	0.08	0.06	**0.10 (0.05)**	0.02	0.08	**0.02 (−0.02)**	−0.09	0.13^*^	–

Correlations across measures, but within a given motive domain are highlighted in boldface. Bipartial correlation coefficients between PGI, PRF/IPQ and PSE with the shared variance within each construct (PGI, PRF, or PSE) partialled out are given in parentheses.

* p < 0.05;

** p < 0.01;

*** p < 0.005.

### Relationships of implicit motives with self-attributed motives and personal goals

Our results lend support to Hypothesis 1, that is, that within a given domain the implicit motive measure (PSE) we found no significant relationship with the explicit motive measures for self-attributed motives (PRF/IPQ) and personal goals (PGI): no significant positive correlations emerged between implicit motive scores on the one hand and corresponding self-attributed motive and personal goal scores on the other. In the power domain, a significant negative correlation emerged between PSE motive and PGI goal scores. The only positive correlation is a cross-domain correlation between the PSE achievement motive score and PGI affiliation goal score.

### Relationships between self-attributed motives and personal goals

The results also support Hypothesis 2, that is, that within a given domain the measures for self-attributed motives and personal goal commitments are correlated: significant and substantial positive correlations emerged between PRF/IPQ motive scores and the corresponding PGI goal scores, although effects were weaker in the power domain than in the other two domains.

### Bipartial correlation analysis

Correlations within and between assessment methods (i.e., self-reported motives and goals; content-coded motives) can be biased by method overlap (e.g., self-report response format), but also by fuzzy boundaries between the measured constructs and thus a lack of specificity (e.g., PRF scales of dominance and affiliation as well as IPQ scales of social potency and social closeness tend to correlate positively with each other, because they represent facets of extraversion or positive emotionality; see Costa and McCrae, 1988; Donnellan et al., 2005). However, we were interested in the degree to which, for instance, the self-reported affiliation motive *specifically* correlated with the implicit affiliation motive, net of whatever overlap the self-report measure of affiliation motivation has with self-report measures of dominance and achievement or whatever overlap the implicit affiliation motive has with implicit measures of power and achievement. We therefore conducted bipartial correlation analyses (Cohen and Cohen, 1983) controlling for non-specific variance by partialling each motivational-domain variable for the two other measures that used the same response format, but assessed different motivational domains. Thus, for instance n Affiliation (PSE), partialled for n Power and n Achievement (PSE), was correlated with the self-attributed affiliation motive (PRF/IPQ), partialled for PRF/IPQ measures of achievement and dominance. Table 2 presents the results of this analysis. The results show that, except for the bipartial correlation between the PGI power goal score and PRF/IPQ power motive score, which was non-significant, all bipartial correlations were similar to their simple-correlation counterparts, suggesting that the reported relationships are not due to method variance or measurement specificity problems.

### Within-domain correlation of implicit motives, self-attributed motives, and personal goals by study

Further, the data lend support to Hypothesis 3, i.e., that the relationships proposed in Hypothesis 1 and 2 can be observed in US and German samples alike. We examined the within-domain correlations between PSE motive scores, PRF/IPQ motive scores, and PGI goal scores by study (see Table 3). Within the domains of affiliation and achievement, PRF/IPQ motives scores and PGI goal scores correlated positively in all studies. In the power domain, PRF/IPQ motive scores and PGI goal scores significantly correlated only in Study 3, the German sample, whereas in Study 1 and 2, the American samples, correlations were also positive but non-significant. There were no significant positive correlations between PSE motive scores and PRF motive scores or between PSE motive scores and PGI goal scores in any study. Again, the only significant correlations between PSE and PRF or PGI are negative (between PSE achievement motive score and PRF achievement motive score in Study 3 as well as between PSE power motive score and PGI power goal score in Study 1). We furthermore analyzed whether the relationships are equivalent across studies (and thus cultural background) by testing whether between-measure slopes [i.e., the slope for n Affiliation (PSE) and affiliation goal commitment] were moderated by study. Using Bonferroni correction with a *p*-value of 0.006 (0.05/9) to adjust for α-error accumulation in multiple analyses, no significant influence of study on the relationships between motivational measures was found.

**Table 3:** **Within-domain correlations between personal goal commitments (PGI), self-attributed motives (PRF/IPQ), and implicit motives (PSE) for studies 1 and 2 (US samples) and 3 (German sample)**.

**Domain**	**PRF/IPQ × PGI**	**PSE × PGI**	**PSE × PRF/IPQ**
**POWER**			
Study 1	0.16	−0.13	−0.02
Study 2	0.09	−0.24^*^	0.04
Study 3	0.21^*^	0.01	−0.03
**ACHIEVEMENT**			
Study 1	0.27^**^	0.06	0.04
Study 2	0.33^***^	0.01	0.17
Study 3	0.32^***^	0.06	−0.25^*^
**AFFILIATION**			
Study 1	0.22^*^	0.01	−0.08
Study 2	0.21^*^	0.16	0.05
Study 3	0.37^***^	0.12	0.07

* p < 0.05;

** p < 0.01;

*** p < 0.005.

## Discussion

The aim of the present research was to shed further light on the relationships between implicit motives, self-attributed motives, and personal goals by reanalyzing three empirical studies in which measures of implicit motives, explicit motives, and ideographically assessed explicit goal commitments had been administered. Based on McClelland et al.'s (1989) two-system model of motivation, we proposed that personal goals and self-attributed motives are parts of the same motivational system and should therefore be more strongly correlated with each other within a given motivational domain than with corresponding implicit motive measures, which belong to another motivational system. Further we assumed that these relationships would be similar in US and German research participants. The results we obtained in 309 research participants in the US and in Germany support these propositions:

First, correlations between implicit motives on the one hand and personal goals or self-attributed motives were generally low and non-significant. Any significant overlap between implicit and explicit motive measures was either negative or across different domains. These findings support McClelland et al.'s (1989) proposition that implicit and explicit motive measures tap into two distinct and independently operating behavior-regulating systems (see McClelland et al., 1989; Schultheiss, 2008).

Second, personal goal commitments and self-attributed motives were positively and significantly correlated across all motivational domains, a finding that is consistent with McClelland et al.'s (1989) claim that goals and self-attributed motives are both representatives of the explicit system. However, compared to the domains of achievement and affiliation, correlations within the power domain were lower and no longer reached statistical significance when we computed bipartial correlations, which controlled for method variance and construct specificity. This might be due to the fact that for the assessment of commitment to power goals the instructions contain independence and social influence but not dominance, the PRF's predominant power aspect. Hence, future research should render the description of power goals more similar to semantic content of the PRF dominance scale to test whether this enhances the variance overlap. Moreover, with an average *r* of 0.24, correlations between measurements of personal goals and self-attributed motives were moderately high. This suggests that self-attributed motives and personal goals are related yet distinct constructs, an interpretation that is consistent with the view of explicit motives as abstract, enduring values and personal goals as more concrete, flexible, and time-limited instantiations of these values (e.g., Hofer et al., 2010a,b). The magnitude of the correlations between explicit motives and goals we observed in our data is very comparable to the correlations reported by others (e.g., Emmons and McAdams, 1991; King, 1995).

Overall, the described pattern of relationships was comparable for US and German samples. However, for the domain of power the predicted correlation between personal goal commitments and self-attributed motives was found in the German, but not in the US-American samples. We speculate that US Americans construe power goals in a way that is more distinct from a general self-attributed dominance orientation than Germans do.

### Limitations and future directions

A shortcoming of this study is that different instruments have been used across the studies to measure explicit motives which can impair the comparability of the results. However, because we found analogous result patterns across studies, we assume that this had a negligible impact on the data. Moreover, to ascertain the robustness of the findings, it would be desirable to replicate them using more than one type of implicit-motive measure. The options for a broader assessment of implicit motives are currently limited by the lack of well-validated alternatives to the PSE. An exception to this rule may be variants of the Implicit Association Test for which encouraging validation results have been reported for the domains of achievement (Brunstein and Schmitt, 2010) and power (Slabbinck et al., 2013). Finally, we would like to draw attention to the idea that a lacking statistical relationship does not necessarily imply unrelated constructs. As Thrash et al. (2012) have stressed, lacking statistical overlap can stem from actual divergent validity of the constructs, but also from methodological shortcomings, such as unreliable measurements, which can artificially lower the actual overlap of the constructs. We have addressed the latter concern to some extent by using bipartial correlation analyses to control for non-specific method variance in the present research. Future research should address this issue by systematically testing the effects of various forms of measurement error as potential factors leading to the inflation or attenuation of correlations between implicit and explicit motivation measures.

## Conclusion

The present findings add to a growing literature suggesting that there is no significant relationship between PSE measures of implicit motives on the one hand and self-report measures of motives and personal goals on the other (e.g., Spangler, 1992; King, 1995; Schultheiss and Brunstein, 2001; Pang and Schultheiss, 2005). With regard to the relationship between implicit motives and explicit goals, our findings confirm observations by King (1995); Brunstein et al. (1998); Schultheiss et al. (2008a), and Hofer et al. (2010a,b), which all suggested very low variance overlap between measures of these constructs. They stand in marked contrast to Emmons and McAdams' (1991) finding that implicit motives and personal goals are substantially and positively correlated. It could be argued that differences in methods may account for the differences in results between their study and ours: Emmons and McAdams content-coded freely generated lists of goals for motivational imagery; we had participants list one goal for each motivational domain and rate their commitment to the goal. However, this argument is weakened by King's (1995) findings, who used the same methods as Emmons and McAdams (1991) but applied them to a larger sample and, like us, failed to observe substantial variance overlap between implicit motive and goal striving measures. This suggests that the goals people adopt are related to their self-attributed motives, but statistically independent of their implicit motives. Nevertheless, peoples' goals may interact with their implicit motives in shaping outcomes such as emotional well-being (see Brunstein et al., 1998; Schultheiss et al., 2008a; Hofer et al., 2010a,b). In sum, our results support McClelland et al.'s (1989) notion of two distinct and autonomously operating implicit and explicit motivational systems.

### Conflict of interest statement

The authors declare that the research was conducted in the absence of any commercial or financial relationships that could be construed as a potential conflict of interest.

## Footnotes

^1^Evidence for the criterion validity of the PGI as well as the PSE comes for instance from a study in which these measures jointly predicted emotional well-being and depression (Schultheiss et al., 2008a).
